# Comparative assessment of SNPs and microsatellites for fingerprinting, parentage and assignment testing in species of *Eucalyptus*

**DOI:** 10.1186/1753-6561-5-S7-P41

**Published:** 2011-09-13

**Authors:** Leonardo Correia, Danielle Faria, Dario Grattapaglia

**Affiliations:** 1 EMBRAPA Genetic Resources and Biotechnology – Estação Parque Biológico, 70770-910, Brazilia, DF and Genomic Sciences Program - Universidade Católica de Brasília - Brazilia, Brazil; 2 EMBRAPA Genetic Resources and Biotechnology – Estação Parque Biológico, 70770-910, Brazilia, DF, Brazil

## Background

Brazil stands on the international scenario by having one of the largest natural forest heritage and extensive sustainable planted forests. Brazilian planted forestry is based mainly on fast growing eucalyptus and pines, with the pulp, paper and steel industries as the major consumers. Introduced commercially in Brazil in the early twentieth century, the eucalypts have experienced increasing levels of genetic improvement over the years. Along with the classical breeding, the use of genetic markers has positively influenced breeding programs, contributing to quality control processes of clonal forestry and advanced breeding. Microsatellite markers have been the main tool used to date. They are multiallelic, highly polymorphic and thus very efficient for several applications that require identification and discrimination of elite clones and determination of parentage. While di and tri-nucleotide repeat microsatellites tend to be more polymorphic, tetra and higher order repeats allow a more robust allele calling [1]. SNP markers genotyped by a high-throughput system have been recently developed for Eucalyptus [2] but not yet routinely incorporated in Eucalyptus breeding programs. In spite of their lower genetic information content, many more SNPs can be simultaneously typed using automated systems, an appealing feature to operational breeding programs. The aim of this study was to comparatively evaluate the resolving power and precision of different sets of molecular markers (microsatellites and SNPs assayed by the Golden Gate technology) for the most common operational applications in *Eucalyptus* breeding programs.

## Methods

Samples belonging to six species of Eucalyptus (*E. grandis*, *E. urophylla*, *E. globulus*, *E. nitens*, *E. camaldulensis and E. dunnii*) were genotyped with three different sets of markers: (1) 24 di- and trinucleotides microsatellites (2) 17 tetra-, penta- and hexanucleotides microsatellites and (3) selected subsets of SNPs from the 768 developed earlier [2]. Microsatellites were genotyped by capillary electrophoresis in an ABI3100 genetic analyser and data collected under dye set D using Genescan/Genotyper.SNP genotyping was performed using an Illumina BeadStation 500 GX at the Genome facility of the University of Florida and the data analyzed using the software GenomeStudio. Multilocus genotypes were obtained with the two microsatellite panels and a set of the 96 most informative SNPs for individual identification based on an algorithm that takes into account minimum allele frequency and observed heterozygosity within and across species. The genotype data were used to estimate the combined probability of paternity exclusion (PE) and probability of identity (PI) for the marker panels. Additionally, markers were assessed for their ability to estimate genomic ancestry of individuals, a particularly useful application for determining the origin of spontaneous Eucalyptus hybrids. All typed individuals were assigned probabilistically to a given number of populations inferred with a Bayesian approach without any prior population information using the STRUCTURE package [3]. The analyses were performed with each set of microsatellites and with a specific subset of 96 ancestry informative SNPs, selected with an algorithm that focused on SNPs that display contrasting allele frequencies among species.

## Results

The panel of di- and trinucleotide microsatellites showed the fastest increase in the combined paternity exclusion with the addition of new markers. For example, a PE > 99% was achieved with only five markers in *E. grandis*. The panel of tetra-, penta- and hexanucleotide microsatellite was unable to reach a PE of 99% even with all 17 markers were used. The panel of 96 SNPs showed a slower increase in the combined paternity exclusion with the addition of new markers, but needed only 35 markers to reach a PE > 99%. The same trend was observed with the estimate of probability of identity. In the analysis of genetic structure, all three sets of markers correctly inferred the six genetically distinct populations, corresponding to the six species studied. Furthermore, both panels assigned correctly all individuals to their respective species. The SNP panel showed the highest average proportion of correct assignment for all six species, followed by the di- and trinucleotides and at last the tetra-, penta- and hexanucleotide panel, although these estimates were not statistically different between the three marker panels tested, (p= 0.327).

## Conclusions

Results show that di- and trinucleotide microsatellites tested are around three times more informative than the tetra-, penta- and hexanucleotide microsatellites used and about seven times more informative than the specific SNPs employed for assessment of parentage and individual identification. Although more informative, the di- and trinucleotide microsatellites are subject to a higher inaccuracy in allele calling, which can result in inconsistencies across analyses carried out in different labs.All three marker panels showed high resolution power to detect genetic structure and carry out assignment tests in species of *Eucalyptus*. We are now extending this analysis to controlled and spontaneous hybrids to compare the estimates of genomic ancestry provided by the three marker panels. Additionally we are developing much larger sets of SNPs by re-sequencing reduced genomic representations of pooled individuals from all six species. This should allow a targeted selection of more ancestral SNPs that are homogeneously polymorphic across species to be used for fingerprinting and parentage or historically more recent SNPs that display contrasting allele frequencies among species and thus be more useful for genomic ancestry determination. We anticipate that despite the lower individual genetic information content, careful selection of SNPs panels for targeted applications coupled to their accurate genotype calling and high throughput will become an increasingly attractive alternative for operational application in breeding programs.
